# Development of Optical-Guiding Scintillators with Ultrafine (~12 μm) Uniform Scintillator Cores for High-Resolution X-Ray Imaging

**DOI:** 10.3390/ma19091834

**Published:** 2026-04-29

**Authors:** Kei Kamada, Masao Yoshino, Yuhei Nakata, Testuo Kudo, Yoshiyuki Usuki, Naoko Kutsuzawa, Kyoung Jin Kim, Rikito Murakami, Satoshi Ishizawa, Akira Yoshikawa

**Affiliations:** 1 New Industry Creation Hatchery Center, Tohoku University, 6-6-10 Aoba, Aramaki, Aoba-ku, Sendai 980-8579, Miyagi, Japan; masao.yoshino.a5@tohoku.ac.jp (M.Y.); kjkim72@c-and-a.jp (K.J.K.); akira.yoshikawa.d8@tohoku.ac.jp (A.Y.); 2 C&A Corporation, 1-16-23 Ichibancho, Aoba-ku, Sendai 980-0811, Miyagi, Japan; t_kudo@c-and-a.jp (T.K.); usuki@c-and-a.jp (Y.U.); kutsuzawa@c-and-a.jp (N.K.); rikito.murakami.d4@tohoku.ac.jp (R.M.); satoshi.ishizawa.a2@tohoku.ac.jp (S.I.); 3 Institute for Material Research, Tohoku University, 2-1-1 Katahira Aoba-ku, Sendai 980-8577, Miyagi, Japan; nakata.yuhei.q4@dc.tohoku.ac.jp; 4 Department of Materials Science, Graduate School of Engineering, Tohoku University, 6-6 Aoba, Aramaki, Aoba-ku, Sendai 980-8579, Miyagi, Japan

**Keywords:** X-ray imaging, composite materials, scintillator materials, optical fibers

## Abstract

**Highlights:**

- The hollow-fiber process enabled bundled OCS with ultrafine (~12 μm) uniform cores.
- Tl:CCI melt infiltration achieved defect-suppressed core formation.
- Bundled OCS outperformed Tl:CsI in spatial resolution and image contrast.
- Optical-guiding architecture reduced lateral light scattering effects.

**Abstract:**

We report the development of bundled optical-guiding crystal scintillators (OCSs) with ultrafine and uniform scintillator cores (~12 μm) for high-resolution X-ray imaging. Conventional OCS fabrication using iodide scintillators often suffers from iodine volatilization, bubble formation, and core discontinuities, which limit structural uniformity and device reliability. To address these limitations, a hollow-fiber-based fabrication strategy was introduced. Hollow glass fibers were first bundled and drawn without scintillator materials, followed by capillary infiltration of a Tl-doped Cs_3_Cu_2_I_5_ (Tl: CCI) melt. This approach enabled the stable formation of densely packed bundled OCS structures with uniform core diameters of 10–12 μm while suppressing volatilization-induced defects. Radioluminescence measurements confirmed a broad emission peak at ~442 nm, consistent with Tl:CCI scintillation. X-ray imaging experiments demonstrated superior spatial resolution and image contrast compared with a commercial CsI:Tl columnar scintillator. The bundled OCS exhibited an average contrast transfer function (CTF) of 30.7% at ~10 lp/mm, exceeding the reference value. These results demonstrate that the hollow-fiber architecture provides an effective route toward scalable ultrafine-core scintillators and highlight the potential of Tl:CCI-filled OCSs for next-generation high-resolution X-ray imaging.

## 1. Introduction

X-ray imaging is widely used in medical diagnostics, industrial inspection, and security applications [1,2,3,4,5]. Continued improvements in spatial resolution are required for emerging applications such as microstructural analysis and synchrotron-based imaging [6,7,8,9,10]. Traditional scintillators such as Gd_2_O_2_S [11,12,13] sintered sheets and columnar Tl:CsI [14,15] are widely used in flat-panel detectors and CT systems. However, their spatial resolution is limited by lateral light scattering and grain boundaries. Thin single-crystal plates of Gd_3_Al_2_Ga_3_O_12_ [15,16,17,18,19,20,21] have recently enabled submicron resolution imaging. Scintillator thin-film imaging, in which several micron-thick layers are deposited onto substrates, has also been proposed as a method for achieving high-resolution X-ray detection [22,23]. However, their sensitivity is low owing to reduced thickness, creating a trade-off between resolution and detection efficiency. Structured scintillators with optical-guiding architectures have been developed to overcome these limitations. Directionally solidified eutectic (DSE) systems such as Ce:GdAlO_3_/α-Al_2_O_3_ and other eutectic scintillators have demonstrated high-resolution imaging by confining scintillation light within high-refractive-index fibers embedded in a lower-index matrix [24,25,26,27]. However, the scalability and uniformity of DSE structures are constrained by instabilities at the solid–liquid interface during crystal growth, making mass production difficult. Imaging plates fabricated by filling Tl:CsI into micro-patterned silicon substrates have also been proposed, and simulations have demonstrated that an excellent spatial resolution can be achieved under ideal conditions [28,29,30,31]. However, in practical material fabrication, it is challenging to produce thick scintillator layers, and crystal quality remains a significant issue.

Therefore, a novel class of structured scintillators, called optical-guiding crystal scintillators (OCSs), has been proposed [32,33,34]. OCS fibers consist of a high-refractive-index halide single-crystal core and glass cladding, enabling the total internal reflection of scintillation light, similar to that of optical fibers. This configuration allows for scalable fabrication using glass drawing techniques, with core diameters tunable from several hundred microns down to ~10 μm, directly translating into improved spatial resolution. Among candidate materials, Tl-doped CsI (Tl:CsI) [35,36] and Tl-doped Cs_3_Cu_2_I_5_ (Tl:CCI) [37,38] have emerged as promising scintillator cores due to their high light yield, density, and compatibility with glass cladding. Tl:CsI offers a light yield of ~55,000 photons/MeV, a density of 4.51 g/cm^3^, and an effective atomic number of 54, making it highly sensitive to X-rays and γ-rays. Tl:CCI, on the other hand, provides an even higher light yield of ~98,200 photons/MeV, a density of 4.53 g/cm^3^, and a decay time of ~840 ns. The lower melting point of CCI (386 °C) compared with CsI (641 °C) reduces thermal stress during melt infiltration and suppresses iodine volatilization during processing. In addition, the higher light yield of Tl:CCI enhances the scintillation photon output, which contributes to improved signal intensity and image contrast in the OCS-based imaging system. Both materials exhibit refractive indices significantly higher than those of borosilicate or quartz glass, thereby ensuring efficient waveguiding. Recent studies have demonstrated the successful fabrication of OCS fibers using these materials. For Tl:CsI, recrystallization via the Bridgman method has been shown to eliminate voids and polycrystalline domains, resulting in transparent single-crystal cores with enhanced optical-guiding performance. Bundled OCS plates composed of hundreds of such fibers have achieved core diameters as small as 20 μm, with aligned crystallographic orientations and no internal voids [34]. Scintillators that employ refractive-index contrast to form optical waveguiding structures, similar to OCS, have also been reported [39,40]. Considering the refractive indices of the CsI scintillator (n = 1.79) and a representative borosilicate glass (n ≈ 1.47), the scintillation light generated within the CsI core undergoes total internal reflection at the glass–core interface at a critical angle of approximately 55°. OCSs confine scintillation photons within high-refractive-index cores embedded in a lower-index matrix, enabling the suppression of lateral light scattering and improved spatial resolution.

Despite recent progress in structured scintillators, the fabrication of OCSs with ultrafine and uniform cores remains challenging. In conventional iodide-based OCS fabrication, iodine volatilization, bubble formation, and core discontinuities often occur during fiber drawing, making it difficult to obtain dense and uniform structures for high-resolution X-ray imaging. To address this issue, we adopted a hollow-fiber-based fabrication strategy, in which hollow glass fibers are first fabricated and bundled, followed by melt infiltration and crystallization of the scintillator cores. This study presents this improved fabrication process for bundled-type OCSs that enables the stable production of structures with finer cores. The imaging performance of the fabricated OCSs is systematically evaluated and discussed.

Despite recent progress in structured scintillators, the fabrication of optical-guiding crystal scintillators (OCSs) with ultrafine and uniform cores remains challenging. In conventional iodide-based OCS fabrication, iodine volatilization, bubble formation, and core discontinuities often occur during fiber drawing, making it difficult to obtain dense and uniform structures for high-resolution X-ray imaging. To address this issue, we adopted a hollow-fiber-based fabrication strategy, in which hollow glass fibers are first fabricated and bundled, followed by melt infiltration and crystallization of the scintillator cores. Using this approach, bundled OCS structures with ultrafine (~10–12 μm) Tl:CCI cores were fabricated, and their X-ray imaging performance was evaluated.

## 2. Materials and Methods

### 2.1. Fabrication of OCS

**Figure 1:**
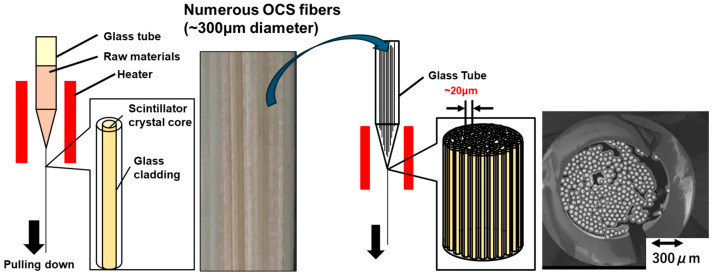
Schematic illustration of the fabrication process for OCS fibers and bundled-type OCS structures.

In the conventional method for fabricating OCS, as previously reported [34], the scintillator raw material is filled into a borosilicate glass container with an inner diameter of 15 mm and a softening point of approximately 800 °C. After the glass tube is evacuated using a rotary pump, Ar gas is introduced into the tube. The container is then heated to its softening point using a heater, causing the simultaneous softening of the glass and melting of the scintillator. Subsequently, the bottom of the glass container is drawn downward at a rate of 2 cm/h. After the diameter of the container was reduced to the designated value, the molten scintillator inside the glass solidifies. As a result, OCS fibers containing scintillator core crystals within glass, with diameters reduced to approximately 300 μm, are successfully fabricated (Figure 1, left). Thousands of OCS fibers are then bundled and inserted into a borosilicate glass container with an inner diameter of 15 mm. Following the same procedure as that used for the fabrication of individual OCS fibers, the assembly is heated under an argon atmosphere and then drawn downward from the bottom of the glass container. Consequently, a bundled-type OCS structure is produced, in which each core is reduced to several tens of micrometers in diameter (Figure 1, right).

**Figure 2:**
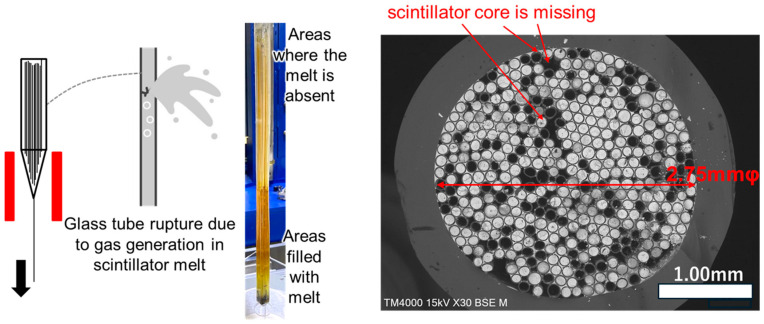
(**Left**) Schematic illustration showing fabrication challenges in bundled-type OCS production. (**Right**) BEI of a cross-sectioned OCS fiber; dark regions indicate missing scintillator cores.

In conventional OCS fabrication processes, iodine from iodide-based scintillators tends to separate as a gaseous component during the production of individual fibers and bundled OCS structures. This separation leads to bubble formation within the molten scintillator core inside the glass cladding, resulting in fiber rupture or melt leakage (Figure 2, left). Consequently, regions lacking scintillator cores may appear in the bundled OCS (Figure 2, right panel). In more severe cases, the glass container ruptures, interrupting the fabrication process and potentially damaging the heating system.

**Figure 3:**
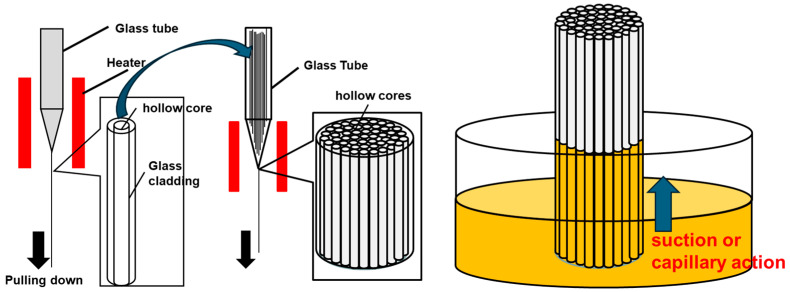
(**Left**) Schematic illustration of the fabrication process for hollow OCS fibers and hollow bundled OCS structures. (**Right**) Schematic diagram showing the filling of the scintillator melt into the hollow-bundled OCS.

To address these issues, improvements in the OCS fabrication process have been investigated. Figure 3 shows schematic illustrations of the improved fabrication process for hollow OCS fibers and hollow-bundled OCSs (left), as well as the filling of the scintillator melt into the hollow-bundled OCS (right). In the revised method, hollow OCS fibers are first fabricated from glass containers without incorporating scintillator material. After bundling a large number of these hollow fibers into a glass container, the assembly is heated and drawn downward in the same manner as in the conventional process, resulting in a hollow-bundled OCS. Because no scintillator melt is present during fiber formation, the fiber diameter can be controlled more precisely, and defects during bundling are minimized. For the crystal growth of the scintillator core, 4N-purity TlI, CsI, and CuI raw materials were used. The starting composition was weighed to yield a nominal composition of (Tl_0.005_Cs_0.995_)_3_Cu_2_I_5_, and the mixed powder was prepared. The hollow-bundled OCS and the raw material powder were loaded into a quartz ampoule. The ampoule was evacuated to approximately 20 Pa using a rotary pump. It was then placed in a three-zone resistance-heating furnace and baked at 100 °C under vacuum to remove residual moisture and air from the ampoule. After baking, the quartz ampoule was heated to 500 °C to melt the scintillator raw materials. The melt infiltration into the hollow cores was carried out by maintaining the ampoule at approximately 20 Pa for 2 h, allowing the molten scintillator to fill the hollow cores by capillary action. After the infiltration process, Ar gas was introduced into the ampoule until atmospheric pressure was reached. The filled structure was then crystallized by slow cooling to room temperature over 20 h under a temperature gradient of 5.5 °C/mm in the three-zone resistance-heating furnace. Finally, a fully filled bundled OCS structure is obtained. The fabricated OCS structure was characterized through scanning electron microscopy (SEM) and backscattered electron imaging (BEI) using a electron microscope (S-3400N, Hitachi, Tokyo, Japan). Cross-sectional samples were prepared by cutting and polishing the OCS specimens prior to observation. SEM/BEI observations were performed at an accelerating voltage of 15 kV to evaluate the core structure, packing density, and filling state of the scintillator cores. 

### 2.2. Luminescence and Scintillation Properties

The fabricated bundled OCS samples were cut into 1 mm thick sections and subsequently polished. Radioluminescence (RL) measurements were conducted using an X-ray tube equipped with a silver target operated at 20 kV and 150 mA to determine the emission wavelength. Spectral data were acquired using a charge-coupled device (CCD) camera (iDus420-OE; Andor Technology, Belfast, UK) integrated with a spectrometer (SR-163; Andor Technology, Belfast, UK). The sample and the X-ray source were placed in close contact. Measurements were conducted with a spectral resolution of 0.25 nm and a 10 μm entrance slit. Data were acquired with an integration time of 60 s, following wavelength calibration using a standard mercury lamp.

### 2.3. X-Ray Imaging Test

High-resolution X-ray imaging was performed using the fabricated bundled OCS. A schematic of the experimental setup is available in a previous study [16]. The imaging system was constructed by integrating the fabricated bundled OCS, a CFI Plan Apochromat Lambda D 10× objective lens (Nikon Solutions Co., Tokyo, Japan), an imaging lens, and a BU-50LN cooled CCD camera (Bitran Corporation, Saitama, Japan) into the AA51 high-resolution X-ray imaging platform developed by Hamamatsu Photonics K.K. (Hamamatsu, Japan). The incident X-rays were first converted into visible light by the scintillator layer. The emitted photons were magnified through the microscope objective and subsequently focused by the imaging lens before being detected by the CCD camera. The system provides an effective field of view of 641 × 481 μm^2^.

To evaluate the imaging performance, comparative tests were carried out using a Tl:CsI columnar scintillator (J6671, Hamamatsu Photonics K.K.) and the OCS. Notably, the OCS lacks a refraction layer. Using a measurement setup similar to that in Ref. [16], the X-ray source used was a TRIX-150LE tube (Toreck Co., Yokohama, Japan) with tungsten target operated at 40 kV with a current of 2 mA and a focal spot size of 1.5 × 1.5 mm^2^. The exposure durations were set to 100 ms and 1 s. A Type9-360 Star test pattern (Moriyama X-ray Equipment Co., Tokyo, Japan) was placed in direct contact with the OCS and Tl:CsI samples. The test pattern featured a lead thickness of 0.05 mm and a minimum spatial frequency of approximately 10 lp/mm. The distance between the X-ray tube and the scintillator was set to 60 mm, and the position of the star chart was controlled using an XY stage.

The transmitted X-rays were converted into visible photons by the scintillator and relayed to the CCD camera via an optical lens system. The contrast transfer function (CTF) was calculated based on the luminescence intensities of the white and black regions of the test pattern image using the following equation:

CTF = (I_max_ − I_min_)/(I_max_ + I_min_) (1)

where I_max_ and I_min_ represent the average intensities of the brightest and darkest areas, respectively. To calculate the CTF, nine line profiles were randomly selected from the region corresponding to 10 lp/mm on the obtained test pattern images.

## 3. Results and Discussion

### 3.1. Fabrication of OCS

**Figure 4:**
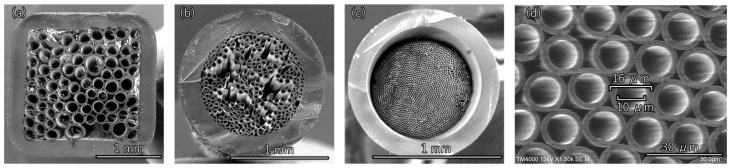
SEM images of hollow bundled OCS. (**a**) Bundled OCS fabricated using hollow OCS fibers with large diameter variation. (**b**) Bundled OCS produced under improper temperature control. (**c**) Hollow-bundled OCS after process optimization. (**d**) Magnified view of the optimized hollow bundled OCS.

**Figure 5:**
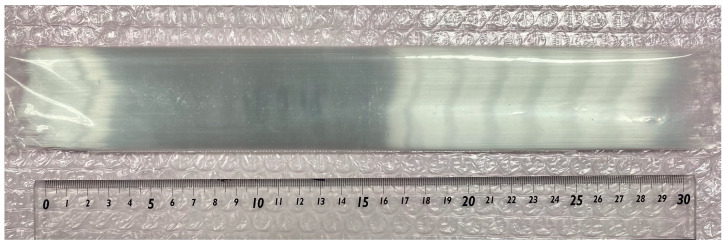
Photograph of a large quantity of hollow OCS fibers produced after optimization of the fabrication conditions.

First, the fabrication conditions for hollow-bundled OCS were optimized. When the dimensional error of the hollow OCS fibers used as the base material for the bundled OCS is large, the core diameters within the bundle become uneven, as shown in Figure 4a, resulting in incomplete packing. Therefore, it is essential to establish a fabrication technique capable of producing hollow OCS fibers with minimal diameter variation. Figure 4b shows an SEM image of a hollow-bundled OCS sample in which voids were formed during the fiber packing process in the glass container and/or where the heating temperature was improperly controlled during fabrication. Techniques for densely packing fibers and precise control of the heating temperature to uniformly soften glass are critical. Following the approach reported in Ref. [34], heater temperature, thermal gradient within the furnace, and drawing speed were optimized, enabling the continuous production of hollow OCS fibers (Figure 5).

For the bundled OCS fabrication, the fibers were cut to a uniform length of approximately 30 cm. Randomly selected fiber samples were measured at both the upper and lower ends using precision calipers, and the results are summarized in Table 1. The diameter variation was found to be within the range of 241–248 μm. The average outer diameter was 243.0 μm with a standard deviation of 1.7 μm, corresponding to a coefficient of variation of 0.7%. This small variation demonstrates that the hollow OCS fibers exhibit a sufficient dimensional uniformity for dense packing during bundled OCS fabrication. Using fibers with uniform diameters, it was possible to pack them densely into a glass container with minimal voids, enabling successful bundling under appropriate temperature conditions. Figure 4c,d show SEM images of the hollow-bundled OCS with densely packed hollow cores. A hollow bundled OCS with an overall diameter of approximately 0.8 mm, consisting of aligned hollow cores with diameters in the range of 10–12 μm, was successfully fabricated.

**Figure 6:**
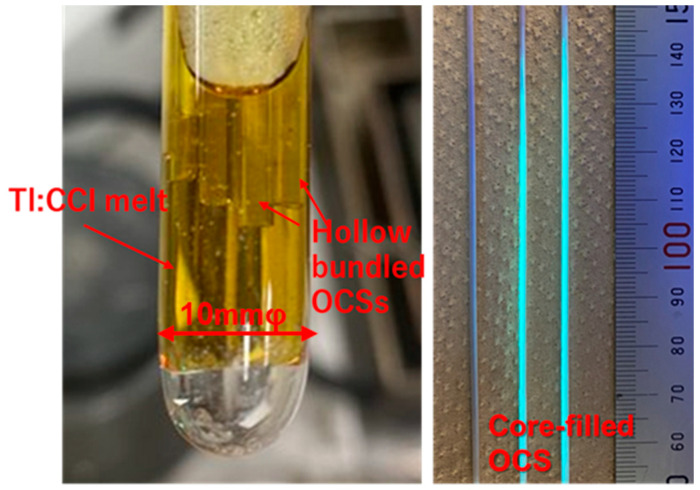
(**Left**): Photograph showing the immersion of hollow-bundled OCS into Tl:CCI melt. (**Right**) Photograph of core-filled bundled OCS under UV irradiation, exhibiting cyan luminescence from the Tl:CCI scintillator.

**Figure 7:**
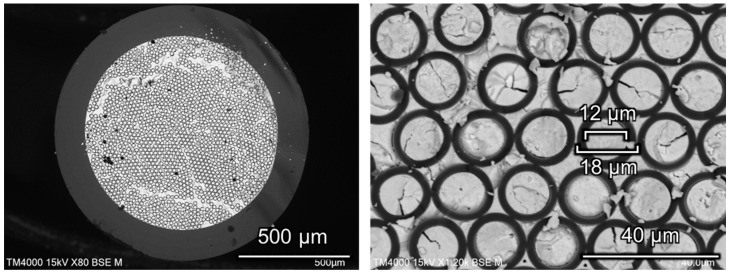
(**Left**): BEI and (**right**) a magnified view of the bundled OCS.

In this study, Tl:CCI scintillators with a higher light output were adopted as the core materials instead of the previously reported Tl:CsI scintillators. The melting point of CCI (386 °C) is lower than that of CsI (melting point: 641 °C) and is reported to be nonhygroscopic, making the fabrication of OCS easier and rendering it a more practical material for applications. As shown in Figure 6 (left), the Tl 0.5%:CCI raw material and the hollow bundled OCS with a core diameter of 10–12 μm (Figure 4c) in the quartz ampoule was heated to 500 °C to melt. The melt infiltration into the hollow cores was carried out by maintaining the ampoule at approximately 20 Pa for 2 h, allowing the molten scintillator to fill the hollow cores through capillary action. After the infiltration process, Ar gas was introduced into the ampoule until atmospheric pressure was reached. The filled structure was then crystallized by slow cooling to room temperature over 20 h under a temperature gradient of 5.5 °C/mm in the three-zone resistance-heating furnace. Figure 6 (right) shows a photograph of the bundled OCS with the filled scintillator cores. Under UV irradiation, the core-filled regions exhibited cyan luminescence originating from the Tl:CCI scintillator. The CCI melt infiltrated more than 11 cm into the cores because of capillary action. As shown in the BEI observation of Figure 7, bundled OCS with an inner diameter of approximately 800 μm was obtained, in which the hollow cores with diameters of 10–12 μm and their surrounding regions were filled with the scintillator

### 3.2. X-Ray-Induced RL and X-Ray Imaging Test

**Figure 8:**
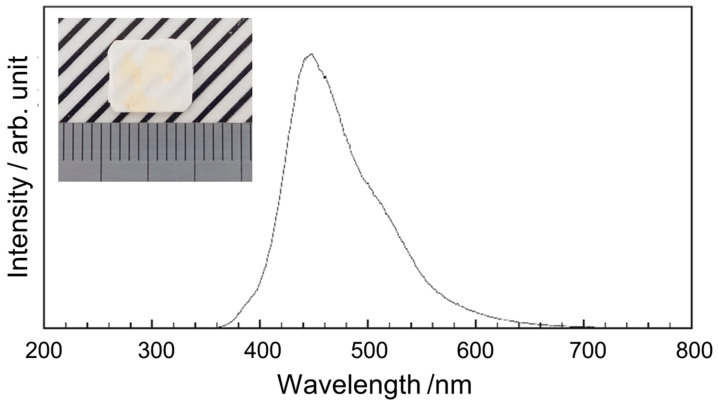
X-ray-induced RL response of the OCS. Inset: photograph of the OCS.

**Figure 9:**
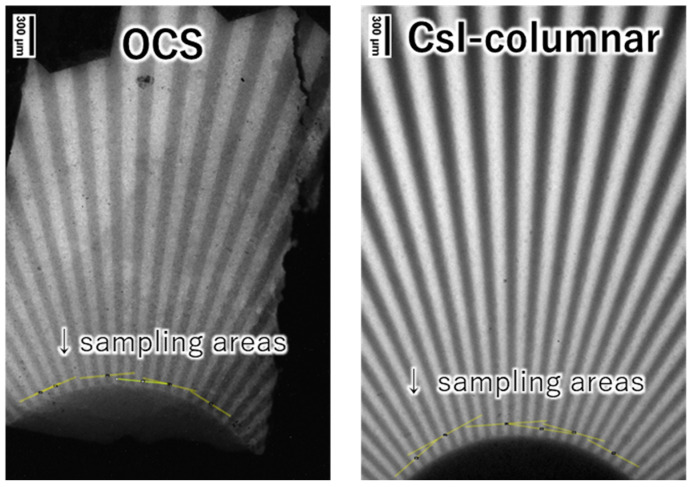
X-ray transmission images of the chart acquired using a 4× objective lens with a 100 ms exposure time: (**left**) OCS and (**right**) Tl:CsI columnar scintillator.

**Figure 10:**
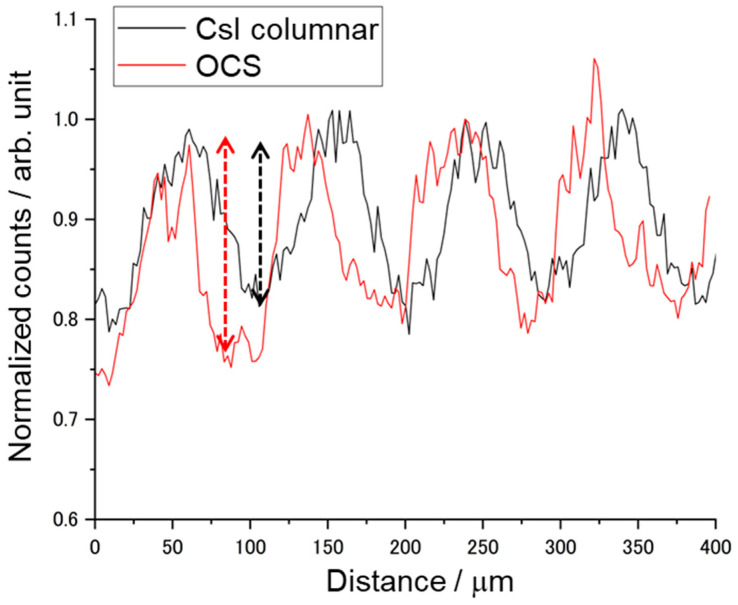
Line profiles comparing the OCS and Tl:CsI columnar scintillator.

The bundled OCS filled with Tl:CCI scintillator cores was cut and polished to a thickness of 0.2 mm, followed by measurements of X-ray-excited RL and X-ray imaging tests. Figure 8 shows a photograph of the sample plate and the measured X-ray-excited RL spectrum. The sample, polished to a thickness of 0.2 mm, exhibited slight optical transparency, despite containing regions with yellow coloration. The yellow coloration observed in some regions is likely associated with point defects or non-stoichiometry formed during melt infiltration and crystallization of the Tl:CCI phase. Possible sources include iodine vacancies, trace impurity contamination, or incomplete crystallization during cooling. Future studies will investigate defect suppression through optimized crystallization, recrystallization and purification processes. A broad emission peak is observed at approximately 442 nm, consistent with the results obtained using a previously reported Tl:CCI scintillator [37,38]. The observed emission can be attributed to self-trapped excitons. Since the RL of the Tl:CCI cores was confirmed, X-ray imaging tests were subsequently conducted for comparison with a commercially available CsI columnar scintillator. Figure 9 shows X-ray transmission images of the chart taken with a 4× objective lens and a 100 ms exposure time for the OCS and Tl: CsI columnar. To maintain consistency, identical settings were applied to the system configuration, sample positioning, and display parameters, including the contrast and brightness. Although the OCS appears slightly dark and exhibits some nonuniform regions, the contrast remains clearly visible. To evaluate the CTF, multiple line profiles were randomly obtained from the region corresponding to 10 lp/mm on the chart, indicated by the yellow lines. An example of the resulting profile is shown in Figure 10. The OCS demonstrated a superior contrast, with more pronounced peak-to-valley differences compared with those obtained using the Tl:CsI columnar scintillator. Table 2 summarizes the CTF values and corresponding spatial frequencies for the six areas. The average CTF across the six points was 30.68% for OCS, exceeding that obtained using the Tl:CsI columnar structure. The imaging measurements were repeated three times, and the average CTF values were calculated. The standard deviation of the CTF values for the OCS sample was approximately 4.8%, confirming the reproducibility of the imaging performance. Regions with CTF values exceeding 40% were also observed. The observed improvement in imaging performance can be attributed to the optical-guiding architecture of the OCS structure. In conventional powder-based or columnar scintillators, scintillation photons undergo multiple scattering events at grain boundaries or column interfaces, which results in a lateral diffusion of the optical signal and the degradation of spatial resolution. In contrast, the refractive index contrast between the Tl:CCI scintillator cores and the surrounding glass cladding enables total internal reflection at the core–cladding interface. This optical-guiding effect confines the scintillation photons within the cores and suppresses lateral light spreading, thereby improving the contrast transfer function (CTF) and spatial resolution. Although the present results demonstrate improved imaging performance compared with a commercial Tl:CsI columnar scintillator, several limitations of the current samples remain. For example, the presence of yellow-colored regions indicates that crystal defects or compositional inhomogeneity may still exist in the scintillator cores. These defects can reduce optical transparency and cause local variations in scintillation intensity. In addition, the uniformity of melt infiltration along the entire fiber bundle may influence the homogeneity of the scintillation response. Further optimization of the crystal growth conditions, including control of the solidification process and reduction in defect formation, will therefore be important for achieving more uniform and higher-quality OCS structures. From a practical perspective, the demonstrated OCS architecture provides a promising pathway for achieving high-resolution X-ray imaging while maintaining sufficient scintillator thickness. Conventional approaches often face a trade-off between spatial resolution and detection efficiency, since thinner scintillator layers improve resolution but reduce X-ray absorption. In contrast, the optical-guiding structure allows the scintillator thickness to be maintained while suppressing lateral light spreading. This characteristic makes OCS structures attractive for applications requiring a high spatial resolution, such as synchrotron-based X-ray imaging, nondestructive testing of microstructures, and advanced medical imaging systems. With further improvements in crystal quality and large-area fabrication, OCS-based scintillators may provide an alternative platform for next-generation high-resolution X-ray detectors.

## 4. Conclusions

In this study, an improved fabrication method for bundled OCS was demonstrated by adopting a hollow-fiber approach. This process effectively overcame the major limitations of conventional methods, including iodine volatilization, bubble formation, and core discontinuities, and enabled the reproducible fabrication of densely packed bundled OCS structures with fine and uniform cores of 10–12 μm. The proposed method provides advantages in terms of scalability and stability compared with those of previously reported fabrication techniques. The use of Tl:CCI as the scintillator core material enabled efficient filling by capillary action and produced RL with a characteristic peak at approximately 442 nm. X-ray imaging tests showed improved imaging performance compared with a reference Tl:CsI columnar scintillator under the present experimental conditions. At the same time, the current samples still contain limitations, including colored defect regions and spatial nonuniformity, which may affect optical transparency and signal uniformity. Therefore, the present results should be regarded as a proof of concept for the hollow-fiber-based OCS fabrication strategy. Further improvements in crystal quality and optical transparency will require more precise control of the solidification process of the scintillator cores. Similar to vertical Bridgman growth, it is important to establish a solid–liquid interface perpendicular to the fiber direction and to promote unidirectional solidification at an optimized crystal growth rate. For practical large-area OCS fabrication, a homogeneous solid–liquid interface must be maintained over the entire OCS structure. This will require a sufficiently large furnace with excellent thermal uniformity, together with optimization of the growth rate and temperature profile. Through these improvements, both a higher crystal quality and larger-area OCS fabrication are expected to be realized. With these advancements, bundled OCS structures filled with Tl:CCI have a strong potential for application in high-resolution X-ray imaging across various fields, such as medical diagnostics, industrial inspection, and synchrotron-based imaging.

## Figures and Tables

**Table 1:** Diameter measurements of randomly selected hollow OCS fibers.

Sample No.	Outer Diameter/μm	
	Top	Bottom
1	244	246
2	244	245
3	243	244
4	246	248
5	241	242
6	241	242
7	242	241
8	244	244
9	241	244
10	243	244

**Table 2:** CTF values and corresponding spatial frequencies for each measurement area.

Sample	Parameter	Area 1	Area 2	Area 3	Area 4	Area 5	Area 6	Average
Hollow-bundled OCS	lp/mm	9.88	10.58	9.66	10.34	10.58	9.88	10.20
	CTF	40.72%	30.64%	34.13%	33.26%	27.65%	27.71%	30.68%
Tl:CsI columnar	lp/mm	11.70	11.40	10.58	10.84	10.58	9.26	10.53
	CTF	22.83%	21.78%	25.03%	23.75%	23.21%	22.63%	23.28

## Data Availability

The original contributions presented in this study are included in the article. Further inquiries can be directed to the corresponding author.
